# Systematic characterization and functional analysis of *trans*-prenyltransferases in *Curcuma wenyujin*

**DOI:** 10.3389/fpls.2025.1712697

**Published:** 2025-11-24

**Authors:** Qian Wang, Yi Su, Tingyu Ye, Shiyi Wu, Huan Liu, Jingwen Sun, Xiu Yin, Tianyuan Hu, Xiaoxia Ma, Shujuan Zhao, Xinde Xu, Xiaopu Yin, Qiuhui Wei

**Affiliations:** 1 School of Pharmacy, Hangzhou Normal University, Hangzhou, Zhejiang, China; 2 School of Life and Environmental Sciences, Shaoxing University, Shaoxing, Zhejiang, China; 3 School of Pharmaceutical Sciences, Shanghai Jiao Tong University, Shanghai, China; 4 Xinchang Pharmaceutical Factory, Zhejiang Medicine Co., Ltd., Shaoxing, China

**Keywords:** *trans*-prenyltransferases (*trans*-PTs), *Curcuma wenyujin*, geranylgeranyl pyrophosphate synthase (GGPS), farnesyl pyrophosphate synthase (FPS), catalytic function

## Abstract

**Introduction:**

*Curcuma wenyujin* (Zingiberaceae), a medicinally significant species within the Curcuma genus, is highly regarded in traditional Chinese medicine for its rich terpenoid constituents. These specialized metabolites serve as the principal bioactive components underpinning the plant's therapeutic effects. As key enzymes in terpenoid biosynthesis, *trans*-prenyltransferases (*trans*-PTs) play crucial roles in regulating metabolic flux.

**Methods:**

*Trans*-PTs in *C. wenyujin* were identified through a comprehensive transcriptome-wide analysis. The identified CwPTs were classified by constructing a phylogenetic tree. Their transcriptional responses to abscisic acid (ABA), methyl jasmonate (MeJA), and gibberellic acid (GA) were assessed. The catalytic functions of CwFPS1 and CwGGPS1 were characterized through *in vitro* enzyme assays and heterologous expression in *Escherichia coli*.

**Results:**

Eight *trans*-CwPTs were identified in *C. wenyujin*. Among them, five CwPTs function as geranylgeranyl pyrophosphate synthases (GGPS), two as solanesyl pyrophosphate synthases (SPS), and one as farnesyl pyrophosphate synthase (FPS). Expression profile assay showed more significant changes in the transcription levels of *Cw*PTs in response to ABA and MeJA than to GA. CwFPS1 catalyzed the biosynthesis of farnesyl pyrophosphate (FPP) through the sequential condensation of dimethylallyl pyrophosphate (DMAPP) and isopentenyl pyrophosphate (IPP), as well as geranyl pyrophosphate (GPP) and IPP. CwGGPS1 catalyzed the condensation of GPP or FPP with IPP to generate geranylgeranyl pyrophosphate (GGPP) *in vitro*. Furthermore, these functions were confirmed in *Escherichia coli*.

**Discussion:**

Our research establishes a molecular foundation for understanding terpenoid biosynthesis in *C. wenyujin*. Future sequencing of the *C. wenyujin* genome will facilitate the development of additional CwPTs.

## 1 Introduction

Terpenoids are the most abundant class of natural products. Many terpenoids serve as active ingredients in traditional Chinese medicine, with significant physiological activity (Chassagne et al., 2019). The biosynthesis of terpenoids originates from two C_5_ basic units: isopentenyl pyrophosphate (IPP) and its isomer, dimethylallyl pyrophosphate (DMAPP) (Roberts, 2007). IPP and DMAPP are produced through either the mevalonate (MVA) pathway in cytoplasm or the 2-C-methyl-D-erythritol-4-phosphate (MEP) pathway in plastids (Bick and Lange, 2003).

Prenyl transferases (PTs), also known as isoprenyl pyrophosphate synthases (IPPS), catalyze the consecutive condensation reactions between IPP and allylic substrates such as DMAPP, geranyl pyrophosphate (GPP), farnesyl pyrophosphate (FPP), geranylgeranyl pyrophosphate (GGPP) etc. to generate a variety of linear pyrophosphate precursors (Liang et al., 2002). Subsequently, these pyrophosphate precursors are acted upon by terpenoid cyclases to form structurally diverse terpenoid skeleton (Gao et al., 2012). According to the difference stereochemical conformations of double bonds resulting from the condensation reactions, PTs are classified into two types: *trans-* and *cis*-PT. PTs serve as the molecular architects governing structural elongation of terpene backbones and driving chemical diversification within terpenoid biosynthesis (Jia and Chen, 2016). Shorter prenyl pyrophosphates (prenyl-PPs) (Cn <50) are synthesized *via trans*-PTs and polyprenyl-PPs (Cn >50) *via cis*-PT in plants (Liang, 2009; Vandermoten et al., 2009). *Trans*-PTs serve as the branch point enzymes in the terpenoid biosynthesis pathway, which include GPP synthase (GPS), FPP synthase (FPS), GGPP synthase (GGPS), geranylfarnesyl pyrophosphate synthase (GFPS), solanesyl pyrophosphate synthase (SPS), and polyprenyl pyrophosphate synthase (PPS) (You et al., 2020). GPS serves as the enzymatic catalyst that orchestrates the condensation between IPP and DMAPP to generate GPP (C_10_), the precursor of all monoterpenoids. FPS catalyzes the sequential addition of IPP to DMAPP or GPP to produce FPP (C_15_), which is subsequently converted into sesquiterpenoids or triterpenoids (Riyadi et al., 2023). GGPS catalyzes the sequential condensation of IPP with DMAPP, GPP, or FPP to form GGPP (C_20_), which is essential for the biosynthesis of gibberellins, chlorophylls, carotenoids, etc (Barja and Rodriguez-Concepcion, 2021). SPS/PPS is responsible for the synthesis of medium- and long-chain isoprenyl-PPs (C_25_-C_50_), such as the side chains of plastoquinone (PQ) and ubiquinone (UQ) (Jun et al., 2004; Wang et al., 2019). In general, *cis*-PTs are considered to catalyze the generation of E-Z mixed polyprenyl-PPs (Cn>50). Currently, the functions of *cis*-PTs remain largely unknown.

An increasing number of amino acid sequences and crystal structures of *trans*-PTs have been characterized (Tarshis et al., 1994). These *trans*-PTs exhibit highly similar amino acid sequences and contain one or two conserved aspartate-rich motifs. The first aspartate-rich motif (FARM) features a core sequence of DDX_2-4_D (X represents any amino acid), and the second aspartate-rich motif (SARM) contains DDX_2_D (Liang et al., 2002). Both motifs are essential for substrate binding and catalytic activity. In Arabidopsis, ten AtGGPSs (GGPPS1-4, 6-11) have been identified as synthesizing GGPP (Beck et al., 2013; Barja and Rodriguez-Concepcion, 2021). For AtGGPPS2, 3, 4 and 11, GGPP is the primary product. *In vitro* experiments indicate that the N-terminal GST fusion of AtGGPPS1 primarily synthesizes GFPP with minimal production of GGPP and GPP. In contrast, GGPP was confirmed as the primary product of the His-tag fusion of AtGGPPS1. AtGGPPS6–11 primarily synthesize GFPP, with GGPP produced as a minor by-product. AtGGPPS12 (At4g38460) is an SSU II protein that lacks GGPPS activity; instead, it interacts with LSU to form a heteromeric GPPS that catalyzes the generation of GPP *in vitro*. GGPPS5 (At3g14510) has been identified as a pseudogene. Additionally, two AtFPPS1-2, two AtSPS1-2, and one AtPPPS have also been identified (Jun et al., 2004; Hsieh et al., 2011). In *Oryza sativa*, twelve *trans*-PTs have been comprehensively analyzed (You et al., 2020). OsGGPPS1 (Os07g39270) is the only identified GGPP synthase. OsGRP (Os02g44780) is an SSU II protein that exhibits no activity; however, it can interact with OsGGPPS1 to enhance its catalytic efficiency. Os01g14630 is a GPS that produces GPP as the sole product from IPP and DMAPP, and generates GGPP from IPP and FPP, but cannot produce FPP from IPP and GPP (Zhou et al., 2017). Three OsSPS1-3 (Os06g46450, Os05g50550, Os12g17320) have been confirmed to synthetize SPP (Ohara et al., 2010; Kim et al., 2017). OsSPS4 is the predicted to be a PPP synthase, while OsFPS1–5 are five predicted FPP synthases.

*Curcuma wenyujin* Y. H. Chen et C. Ling, a plant of Curcuma genus in the Zingiberaceae family, has been cultivated in Zhejiang Province, China, for thousands of years. *C. wenyujin* is recognized as a traditional Chinese medicine plant. Terpenoids are the primary biologically active ingredients in *C. wenyujin*. To date, 137 terpenoid compounds have been isolated from *C. wenyujin* (Li et al., 2021). For instance, elemene (C_15_H_24_), a sesquiterpenoid compound, is the most well-known active ingredient and is used as the anti-cancer drug with no reported severe adverse reactions (Zhai et al., 2019). Therefore, understanding the biosynthetic pathways of terpenoids, particularly the branch point enzymes, is of great significance.

Until now, the *trans*-PTs family of *C. wenyujin* has not been reported. In this study, we identified and analyzed eight presumed *trans*-PTs, including five GGPS, one FPS, and two SPS in *C. wenyujin*. Furthermore, their functions were characterized using a metabolic engineering approach in *E. coli* and enzyme catalysis *in vitro*. Our study reports the *trans*-PTs of *C. wenyujin* for the first time, providing a foundation for exploring a more diverse range of terpenoids.

## 2 Materials and methods

### 2.1 Plant materials, bacterial strains, plasmids

The germplasm of *C. wenyujin* was obtained from Rui’an, Wenzhou, Zhejiang Province, P.R. China, and cultivated in the greenhouse of our laboratory. The cultivation conditions were maintained at 22°C with a photoperiod of 12 hours of light and 12 hours of darkness. The mixed cDNA from the flowers, tender leaves, and tender rhizomes of *C. wenyujin* was used for gene cloning. The *E. coli* strain DH5α was utilized for plasmid propagation, while *E. coli* BL21 (DE3) and BL21star (DE3) were employed for protein expression. The plasmids pMAL-c2x, pET32a, and pBbA5c-MevT(CO)-T1-MBIS(CO, ispA) (https://www.addgene.org/; Number: 35152) were selected as prokaryotic expression vectors.

### 2.2 Bioinformatics analysis

A Hidden Markov Model (HMM) (Accession No. PF00348) based on the polyprenyl_synt domain was applied to identify *trans*-PTs from the transcriptome data. The HMMER module in TBtools v2.052 was employed for sequence retrieval from the publicly available transcriptome dataset downloaded from the Genome Sequence Archive (https://ngdc.cncb.ac.cn/gsa/; accession No. CRA006461), using a stringent E-value cutoff of ≤1e-10. The identified sequences were subsequently validated for the conserved polyprenyl_synt domain using the SMART online platform (http://smart.embl-heidelberg.de/) with default parameters. Multiple sequence alignment was performed using Clustal X version 2.1 to assess sequence conservation. Subcellular localization of the putative *trans*-PTs was predicted *via* WOLF PSORT (https://wolfpsort.hgc.jp/), while theoretical (Mw) and isoelectric points (pI) were computed using the ExPASy Compute pI/Mw tool (https://web.expasy.org/compute_pi/). Phylogenetic reconstruction was conducted in MEGA 11 using the Neighbor-Joining method.

### 2.3 RNA isolation and qRT-PCR assay

RNA was extracted using the FastPure Plant Total RNA Isolation Kit (RC401-01, Vazyme, Nanjing, China), following the manufacturer’s instructions. Subsequently, first-strand cDNA was synthesized with a PrimeScript™ RT Reagent Kit containing gDNA Eraser (R323-01, Vazyme, Nanjing, China). Gene expression quantification was executed on a LightCycler^®^ 96 Real-Time PCR System (Roche, Basel, Switzerland). For gene spatiotemporal expression analysis, flowers, tender leaves and rhizomes, as well as mature leaves and rhizomes, were collected from three independent plants exhibiting consistent growth. For the phytohormone treatments, trefoil-stage sterile seedlings were selected. The seedlings were treated with 250 μM for methyl jasmonate (MeJA), 100 μM abscisic acid (ABA), and 100 μM gibberellic acid (GA), respectively. Tender leaves were then collected for gene expression analysis. All qRT-PCR analyses were performed using three independent biological replicates, with each biological replicate was subjected to three technical replicates. Expression quantification employed the 2^−ΔΔCt^ algorithm normalized against 18S rRNA as the endogenous reference (Livak and Schmittgen, 2001). Statistical validation was achieved through Student’s *t*-test implemented in SPSS Statistics 17.0. Primer sequences for all target genes are listed in **Supplementary Table S1**.

### 2.4 The functional identification of *trans*-PTs in *E. coli*

The open reading frames (ORFs) of CwGGPSs were cloned into the expression plasmid pET32a to construct recombinant plasmid pET32a-CwGGPSs. The plasmid pBbA5c-MevT(CO)-T1-MBIS (CO, ispA) was transformed into BL21star (DE3) to intensify the MVA pathway, resulting in the high-yield FPP strain E0. Subsequently, the recombinant plasmids pET32a-CwGGPSs were respectively transformed into strain E0, yielding the E1 strain. The *ispA* gene was deleted from pBbA5c-MevT(CO)-T1-MBIS (CO, ispA) plasmid to create the modified plasmid pBbA5cΔispA. Additionally, the *ispA* gene in pBbA5c-MevT(CO)-T1-MBIS (CO, ispA) plasmid was replaced with CwFPS1, resulting in the modified plasmid pBbA5cΔispA-CwFPS1. The plasmids pBbA5cΔispA and pBbA5cΔispA-CwFPS1 were then transformed into BL21star (DE3) to produce E2 and E3 strains, respectively. All primers used in this study are listed in **Supplementary Table S1**.

Recombinant strains were incubated in LB liquid media containing 34 µg/mL of chloramphenicol at 37°C with shaking at 250 rpm until the OD_600_ reached between 0.6 and 0.8. Subsequently, 0.5 mM isopropyl β-D-1-thiogalactopyranoside (IPTG) was added to induce protein expression. Simultaneously, 5 ml of n-dodecane was layered on the surface of the bacterial culture. The culture conditions were maintained at 20°C with oscillation at 180 rpm in the dark for 48 hours. After fermentation, the culture was centrifuged, and the n-dodecane layer was collected and filtered through a 0.22 μm filter membrane for subsequent gas chromatography-mass spectrometry (GC-MS) analysis.

### 2.5 Protein expression and purification of *trans*-PTs

The ORFs of CwGGPS1–5 and CwFPS1 were cloned using cDNA of *C. wenyujin* as a template, then inserted into the pMAL-c2x vector at a *Bam*HI site. The primers used are listed in **Supplementary Table S1**. BL21(DE3) strains harboring the plasmids pMAL-CwFPS1 or pMAL-CwGGPS1–5 were cultured in 500 mL of LB medium containing 100 µg/mL of ampicillin, respectively. The cultivation conditions maintained at 37°C with shaking at 200 rpm for 14 hours. Protein expression was induced by adding 0.5 mM isopropyl IPTG when the OD600 reached 0.6 to 0.8. The culture was then continued at 20°C with shaking at 180 rpm for 18 hours. Then, the cells were collected by centrifugation at 7000 g for 10 minutes. The collected cells were resuspended in 10 mL of binding buffer (20 mM Tris-HCl, pH 8.0, 300 mM NaCl, 5 mM imidazole) and subsequently disrupted using a high-pressure homogenizer (JNBIO, JN-02C, China). After centrifugation at 8000 g for 45 minutes, the soluble supernatant was collected and added to 5 mL of nickel agarose resin, followed by incubation at 4°C for 2 hours to allow binding. Subsequently, the Ni-protein conjugate was rinsed with 200 ml of wash buffer (20 mM Tris-HCl, pH 8.0, 300 mM NaCl, 30 mM imidazole). Finally, the protein was collected in 50 ml of elution buffer (20 mM Tris-HCl, pH 8.0, 300 mM NaCl, 250 mM imidazole). The collected protein was then concentrated using an Amicon Ultra-0.5 Centrifugal Filter (30 kDa molecular weight cutoff, Millipore, Billerica, MA, USA) and exchanged into cryoprotection buffer (50 mM HEPES, pH 7.5, 10 mM MgCl2, 5 mM DTT, 5%(v/v) glycerol). Sodium dodecyl sulfate-polyacrylamide gel electrophoresis (SDS-PAGE) was performed to detect purified protein.

### 2.6 Enzymatic activity assay *in vitro*

The enzymatic reaction mixture contained 0.2 mg purified enzyme protein in 200 μL reaction buffer (50 mM HEPES, pH 7.5, 10 mM MgCl2, 5 mM DTT, 5%(v/v) glycerol), with 50 μM of either DMAPP/IPP, GPP/IPP, or FPP/IPP as substrates. The enzymatic reaction was carried out at 30°C with shaking at 130 rpm for 3 hours. Subsequently, 6 μL of calf intestinal alkaline phosphatase (CIP) (5,000 units/mL) and 20 μL of 10 × CutSmart buffer (New England Biolabs) were added to the reaction mixture which was covered with 200 μL of n-hexane. The mixture was then incubated at 37°C with shaking at 130 rpm for 2 hours to carry out dephosphorylation. After incubation, the reaction mixture was extracted twice using 200 μL of n-hexane each time, and all the n-hexane layers were collected for further analysis by GC-MS. The substrates DMAPP, IPP, GPP, and FPP, used in enzymatic reactions, were purchased from Sigma-Aldrich.

### 2.7 GC-MS analysis

Agilent 8860 GC system (USA), coupled with a 5977B mass selective detector, was used for product analysis in the heterologous expression experiments conducted in *E. coli*. Agilent 8890 GC system (USA), coupled with a 7000D mass selective detector, was employed for product analysis in enzymatic reaction experiments *in vitro*. Both instruments are equipped with an HP-5 column (30 m×0.32 mm×0.25 μm). A one-microliter sample was injected using a splitless injection method. Helium served as the carrier gas at a constant flow rate of 3.0 mL/min. The oven temperature was initially set at 80°C, followed by a ramp to 180°C at a rate of 40°C/min, where it was held for an additional 2 min. The temperature was then increased to 220°C at a rate of 20°C/min and held for 2 min, before finally rising to 240°C at a rate of 10 °C/min for 1 min. The injector and detector temperatures were set at 260°C and 295°C, respectively. Electron impact (EI) was employed as the ionization source, and the mass spectrometry scanning range was set to 50–500 m/z. Standards of geraniol (GOH, purity ≥ 98%), farnesol (FOH, purity ≥ 98%), and geranylgeraniol (GGOH, purity ≥ 95%) were obtained from Yuanye Bio-Technology Co., Ltd. (Shanghai, China).

## 3 Results and discussion

### 3.1 Identification and clone of *trans-*CwPTs in *C. wenyujin*

A total of eight *trans*-CwPTs were identified from the transcriptome data of *C. wenyujin*. The ORF of the *trans-CwPTs* were cloned using PCR with cDNA from *C. wenyujin* as the template. The amino acid sequences are listed in **Supplementary Table S2**. The number of amino acids in the *trans-*CwPTs ranges from 340 (CwPT5) to 418 (CwPT8). The Mw of the *trans-*CwPTs spans from 36.7 kDa (CwPT5) to 45.9 kDa (CwPT8), and the theoretical pI varies from 5.74 (CwPT6) to 6.65 (CwPT8). CwPT1–5 and CwPT7 were predicted to be localized within the chloroplast, while CwPT6 was predicted to be present in the cytoplasm. In contrast, CwPT8 was predicted to be localized in the mitochondrion. All the characteristics of the identified *trans*-CwPTs are summarized in **Supplementary Table S3**.

### 3.2 Phylogenetic analysis of *trans*-CwPTs

**Figure 1:**
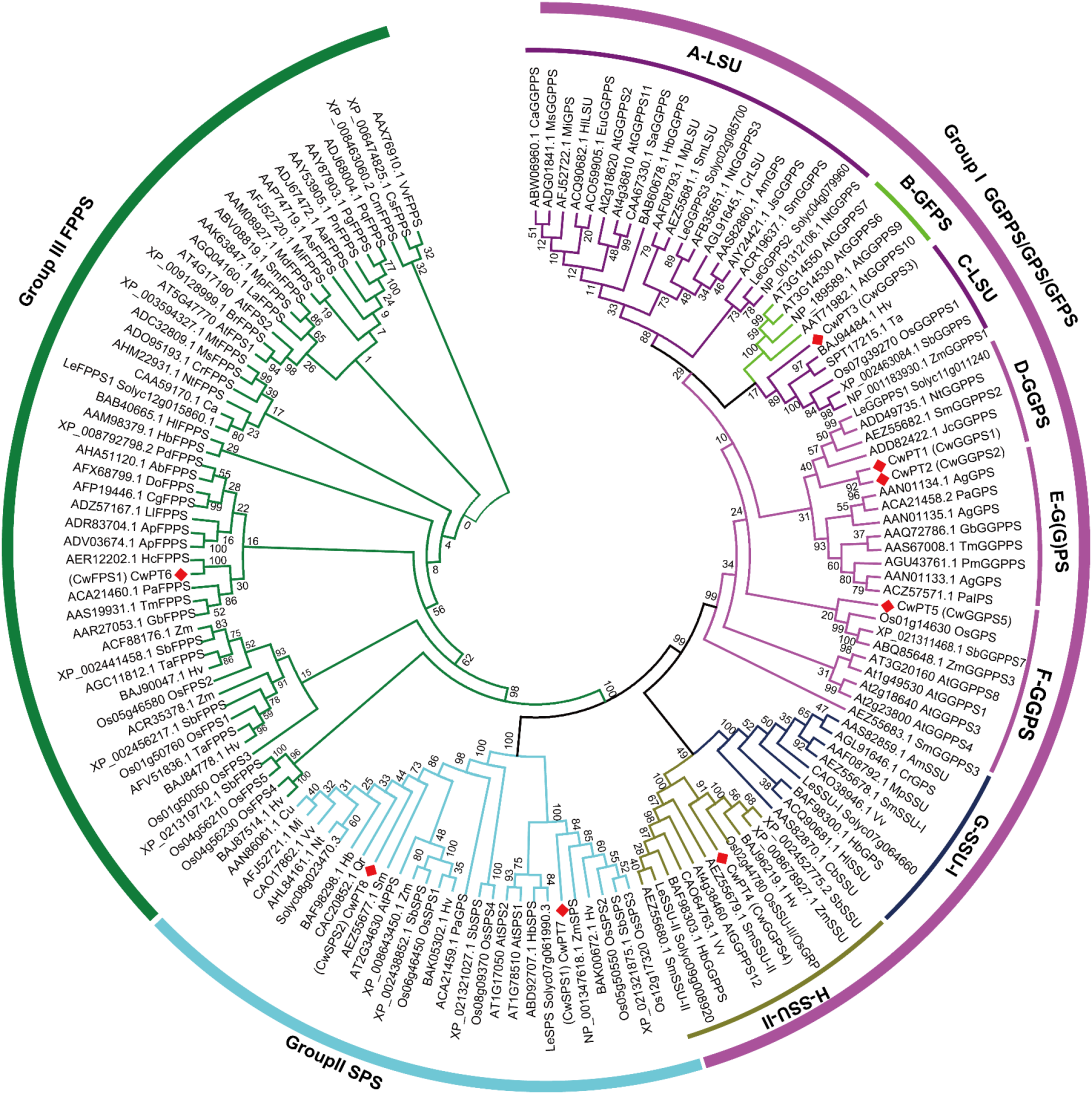
Phylogenetic tree of *trans*-PTs in plants. The 150 *trans*-PTs from *C. wenyujin*, *A. thaliana*, rice, *Lycopersicon esculentum* (tomato), *Hordeum vulgare*, and others were used in the construction of the phylogenetic tree, as listed in **Supplementary Table S4**. Plant *trans*-PTs were named using a combination of accession numbers, species abbreviations, and predicted or confirmed enzyme activities. The phylogenetic tree was constructed using MEGA11 software with the Neighbor-Joining tree method. Bootstrap support for the tree topology was assessed with 1,000 replicates. Different colors represent various groups and subgroups. The red square indicates the *trans*-PTs from *C. wenyujin*.

A phylogenetic tree was constructed using 150 *trans*-PTs from *Arabidopsis thaliana*, *Oryza sativa*, *Solanum lycopersicum*, and other species to analyze their phylogenetic relationships. These *trans*-PTs were classified into three major groups: GGPS/GPS/GFPS, SPS, and FPS (**Figure 1**). Group I was further divided into six subgroups, which include LSU (Subgroups A and C), GFPS (Subgroup B), G(G)PS (Subgroup D), and SSU (Subgroups E and F). The clustering results are consistent with previous research (You et al., 2020; Song et al., 2023). The GFPS subgroup, which is part of Group I, is believed to have originated from GGPS (Liu et al., 2016). Gene duplication and moderate expansion of the GGPS family also contributes to the formation of specific GPS types. There are two types of GPS:homomeric GPS and heteromeric GPS. Homomeric GPS consists of two identical subunits, while heteromeric GPS contains an active large subunit (LSU) and an inactive small subunit (SSU), which includes SSU I and SSU II (Chen et al., 2015).

In the D-G(G)PS subgroup of group I, CwPT1 and CwPT2 are found to be orthologous to LeGGPPS1 (Solyc11g011240), NtGGPPS (ADD49735.1), and JcGGPPS (ADD82422), all of which have been characterized for their GGPPS activity (Orlova et al., 2009; Wang et al., 2010; Zhou and Pichersky, 2020; Barja et al., 2021). CwPT5 is identified as an orthologous protein of OsGPS (Os01g14630), which not only catalyzes the production of GPP from IPP and DMAPP but also produces GGPP from IPP and FPP (Zhou et al., 2017). In the C-LSU subgroup, CwPT3 is closely related to OsGGPPS1 (Os07g39270), the only functional GGPPS known to produce GGPP in chloroplasts (Zhou et al., 2017). ZmGGPPS1 (NP_001183930) has also been reported to exhibit GGPPS activity (Vallabhaneni and Wurtzel, 2009). In the F-SSU-II subgroup, CwPT4 has been identified as orthologous to OsSSUII (OsGRP), a small subunit of heteromeric GPPS. Although OsSSUII (OsGRP) exhibits no enzymatic activity on its own, this protein can enhance the catalytic efficiency and specificity of GGPP production when it interacts with OsGGPPS1 (Zhou et al., 2017). In summary, CwPT1–3 are speculated to possess GGPS activity, while CwPT5 is believed to have either GPS or GGPS activity, and CwPT4 is considered to have no activity.

In addition, CwPT6 is classified into group III (FPS). CwPT6 exhibits a similarity of up to 96% with HcFPPS (AER12202) from *Hedychium coronarium*, which produces FPP from DMAPP and IPP substrates (Lan et al., 2013). PaFPPS (ACA21460) from *Picea abies* also demonstrates FPPS activity (Schmidt and Gershenzon, 2007). Therefore, CwPT6 has been designated as CwFPS1. In group II, CwPT7 is closely related to AtSPS1 (AT1G78510) and AtSPS2 (AT1G17050) from *A. thaliana*, as well as OsSPS2 (Os05g50550) from rice. These enzymes are functional in *Escherichia coli* and yeast, utilizing allylic substrates GPP, FPP, or GGPP to produce solanesol (C_45_) and generating UQ-9, indicating that they encode a solanesyl-PP synthase (Jun et al., 2004; Hirooka et al., 2005; Ohara et al., 2010). CwPT8 clustered with AtGPS1 (AT2G34630), which has been reported to possess GPS activity (Bouvier et al., 2000). However, subsequent research has challenged this conclusion, suggesting that AtGPS1 is a medium/long-chain-length prenyl-PP synthase referred to as AtPPPS1 (Hsieh et al., 2011). Additionally, OsSPS1 (Os06g46450) has also been identified as a solanesyl-PP synthase (Ohara et al., 2010). Based on the evolutionary relationships, CwPT7 and CwPT8 are considered to be SPS. The renamed *trans*-CwPTs and their predicted activities are provided in **Supplementary Table S3**.

### 3.3 Conservative structure of *trans*-CwPTs

**Figure 2:**
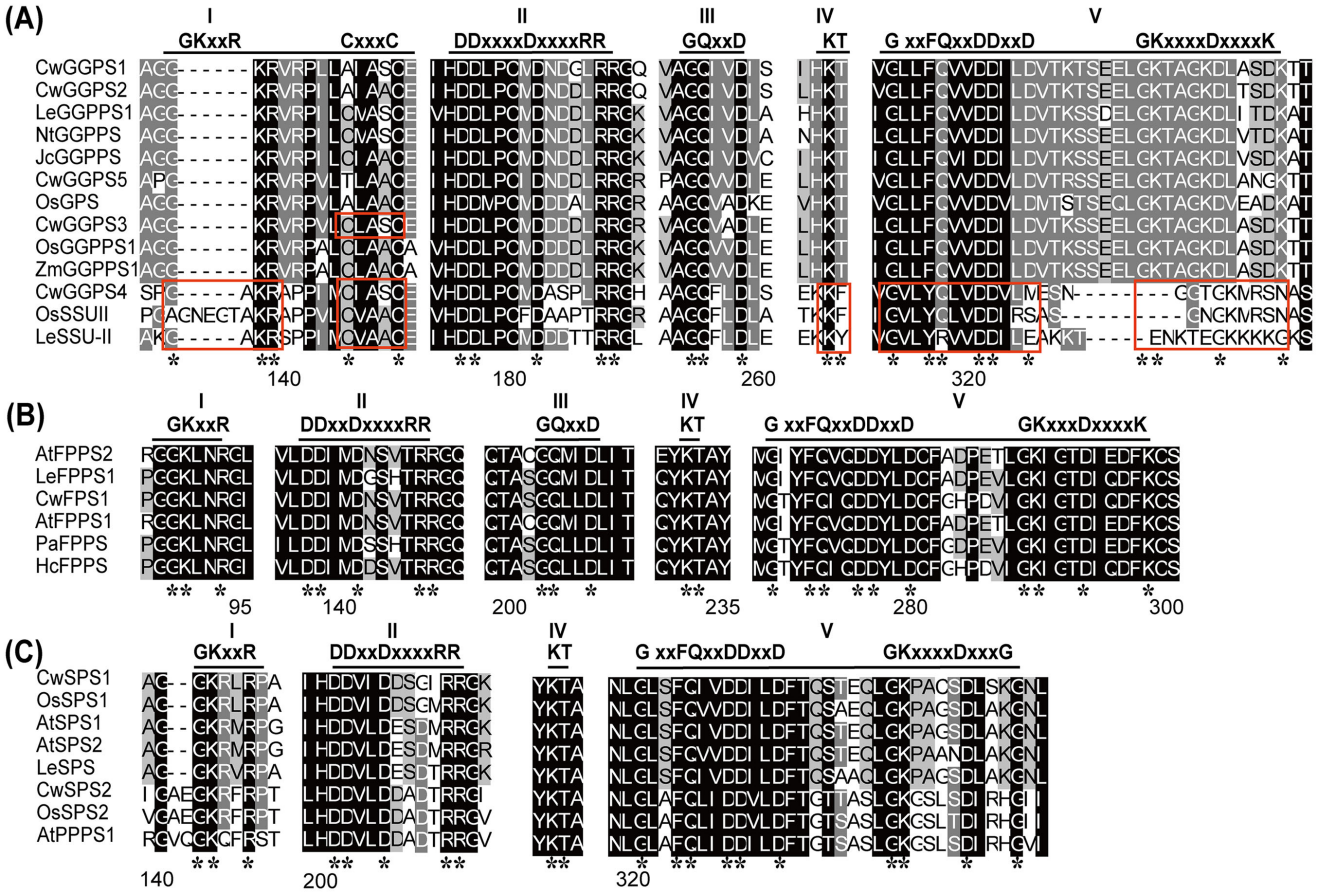
Multiple sequence alignment of *trans*-PTs in plants. The amino acid sequences of the functional *trans*-PTs in groups I **(A)**, groups II **(B)**, and groups III **(C)** were aligned using ClustalX software and visualized with the GeneDoc program. The sequences of conserved motifs are indicated with horizontal lines, while incomplete conserved motifs are highlighted with red boxes. The asterisks indicate conserved amino acid residues within conserved motifs.

Multiple sequence alignments of *trans*-CwPTs and several PTs with known functions from other plants were conducted for each group in the phylogenetic tree to characterize structural conservation (**Figure 2**). Five regions containing conserved motifs were identified in *tans*-PTs. Among them, three distinct catalytic modules, designated as Regions I (GKxxR), Regions II (First Aspartate-Rich Motif, FARM: DDX(2–4)Dx4R), and Regions V (Second Aspartate-Rich Motif, SARM: DDx2D), constitute evolutionarily conserved architectural elements critical for the binding of the allylic substrates and catalysis (Joly and Edwards, 1993; Song and Poulter, 1994; You et al., 2020). Notably, there are several variations in these five regions across different groups. The CxxxC motif in Region I is exclusively found in SSU proteins, as well as in some GGPPS in Group I. SSU interacts with LSU or GGPPS through the motif “CxxxC” to enhance or change their catalytic activity (Wang and Dixon, 2009). For example, the CxxxC motif is present in OsGGPPS1 and OsSSUII, and it has been reported that OsSSUII enhances the catalytic efficiency and specificity of GGPP production when interacting with OsGGPPS1 (Zhou et al., 2017). Therefore, we speculate that CwGGPS4 (SSU II) may interact with CwGGPS3 (LSU) to influence its activity. The GKxxR motif was varies in the SSU II subgroup, with one or more amino acids inserted between G and K. In the SSU II subgroup, Region IV and V are absent. Region V, which contains aspartate-rich motifs (SARM, DDx2D) is necessary for the binding of the allylic substrates and the catalytic activity of PTs (Joly and Edwards, 1993; Song and Poulter, 1994). Consequently, GGPS4 lacks the DDX2D motif and exhibits no catalytic activity (**Supplementary Figures S1**, **S5**), which aligns with this theory.

### 3.4 Expression patterns of *trans*-*CwPTs* in *C. wenyujin*

**Figure 3:**
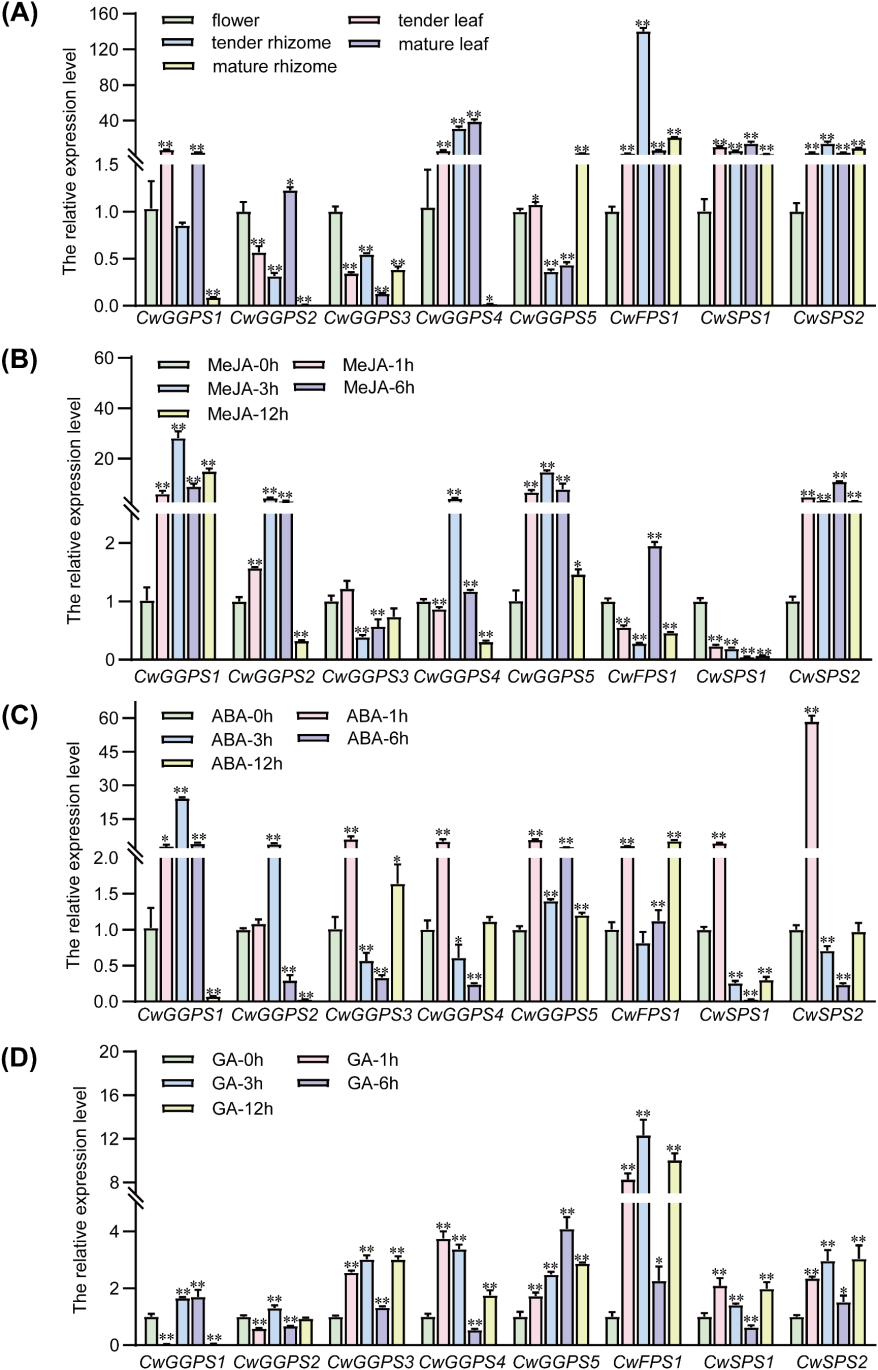
Expression patterns of *trans-CwPTs* in various organs of *C*. *wenyujin* and under hormones induction. **(A)** The expression levels of *trans-CwPTs* in flowers, tender leaves and rhizomes, and mature leaves and rhizomes. **(B-D)** The expression levels of *trans-CwPTs* in the leaves of *C*. *wenyujin* under treatments with MeJA, ABA, and GA. The qRT-PCR method was employed to detect the transcription levels of *trans-CwPTs*, with 18S serving as the internal reference gene. The values were calculated using the comparative 2^−ΔΔCt^ method, where 2^−ΔΔCt^ represents the relative fold-change values compared to the control (flower or 0 h point). Three biological replicates and three technical replicates were performed. Vertical bars represent ± SD (n = 3). A single asterisk indicates *p* < 0.05, and two asterisks indicate *p* < 0.01. Significant differences were defined by a mean fold change ≥ 2 or ≤ 0.5, with *p* < 0.05. A single asterisk indicates *p* < 0.05, and two asterisks indicate *p* < 0.01 (* *p* < 0.05; ** *p* < 0.01).

To profile the expression patterns of *trans*-*CwPT* genes, transcript levels were examined using qRT-PCR (**Figure 3**). The expression levels of *CwGGPS1*, *CwGGPS4*, and *CwSPS1* were relatively high in tender leaves. The *CwFPS1* gene exhibited the highest expression in tender rhizomes, followed by *CwGGPS4* and *CwSPS2*. In mature leaves, *CwGGPS4* displayed the highest expression level, while *CwFPS1* and *CwSPS2* showed relatively high expression in mature rhizomes. *CwGGPS2*, *CwGGPS3*, and *CwGGPS5* were primarily expressed in flowers. The expression pattern of terpenoid biosynthesis genes indicates that terpenoid biosynthesis is not tissue-specific in *C. wenyujin*.

Plant hormones, including MeJA, GA, and ABA, have been reported to influence the biosynthesis of terpenoids in plants (Di et al., 2019; Dong et al., 2022; Yuan et al., 2023; Jiang et al., 2024). After 12 hours of treatment with MeJA, the expression levels of *CwGGPS1* and *CwSPS2* were continuously upregulated in the leaves of *C. wenyujin*. In contrast, the expression of *CwGGPS2*, *CwGGPS4*, and *CwGGPS5* was significantly upregulated initially but then either returned to baseline or decreased. The expression levels of *CwGGPS3* and *CwSPS1* were downregulated throughout the treatment. *CwFPS1* exhibited fluctuating expression levels over the extended treatment period. MeJA typically triggers the biosynthesis of terpenoids, which function as antimicrobial or antiherbivore compounds. This process is mediated through JA signaling cascades that rapidly induce downstream biosynthetic genes (Hua et al., 2021; Cao et al., 2022). The expression patterns of *trans-CwPTs* reflected their JA-specific responses in *C. wenyujin*. Under ABA treatment, all *trans*-*CwPTs* were induced, with *CwGGPS1* and *CwSPS2* exhibiting strong induction within 12 hours. ABA, a key stress hormone, often coordinates abiotic stress responses by enhancing terpenoid production to provide protective functions (Yang et al., 2023; Ye et al., 2024). Most *trans*-*CwPTs* exhibited weak response to GA, excepted for *CwFPS1* within 12 hours. This corresponds to GA’s primary role in promoting growth and development rather than in stress-induced metabolism (Fuentes et al., 2012; Deng et al., 2024).

### 3.5 Heterologous expression and functional identification of CwFPS1 and CwGGPSs in *E. coli*

**Figure 4:**
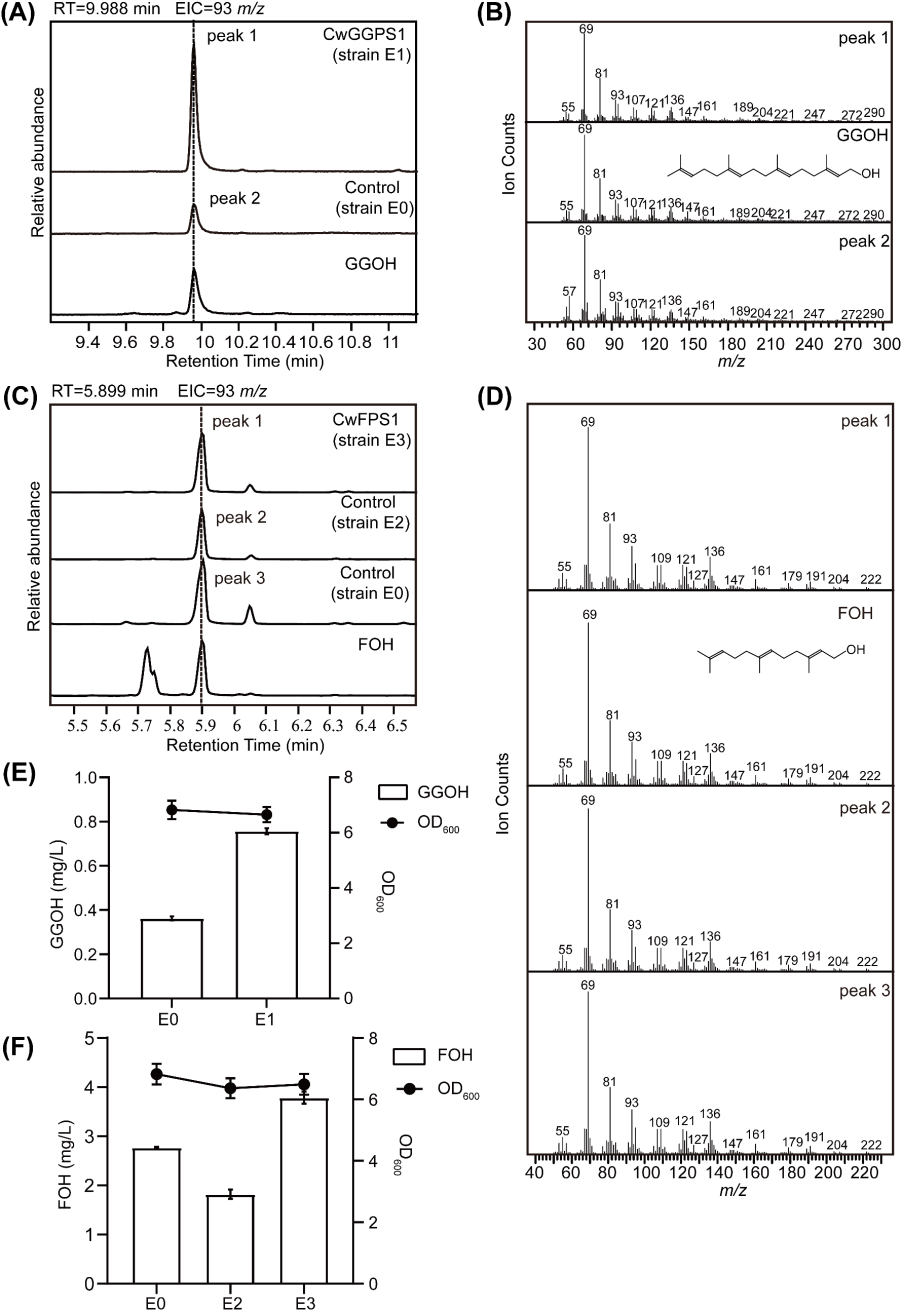
GC–MS analysis of products catalyzed by CwFPS1 and CwGGPS1 in *E*. *coli*. **(A)** GC–MS analysis of the dephosphorylated products. The control strain E0 harbors the pBbA5c-MevT(CO)-T1-MBIS (CO, ispA) plasmid, which contains the FPP synthesis pathway. Strain E1 contains both the pBbA5c-MevT(CO)-T1-MBIS (CO, ispA) and pET32a-CwGGPS1 plasmids. GGOH is the standard chemical compound. **(B)** Mass spectra of peak 1, peak 2, and GGOH. **(C)** GC–MS analysis of the dephosphorylated products. The control strain E2 harbors the pBbA5cΔispA plasmid, which deletes the *ispA* gene. Control strain E0 harbors the pBbA5c-MevT(CO)-T1-MBIS (CO, ispA) plasmid. Strain E3 contains both the pBbA5cΔispA and pET32a-CwFPS1 plasmids. Farnesol (FOH) is the standard chemical compound. **(D)** Mass spectra of peak 1, peak 2, peak 3, and FOH. **(E)** Quantitative analysis of GGOH in strain E1 and control E0, catalyzed by CwGGPS1. **(F)** Quantitative analysis of FOH in strain E3, strain E2, and control E0, catalyzed by CwFPS1.

Plasmid pBbA5c-MevT(CO)-T1-MBIS (CO, ispA) was transformed into BL21star (DE3) to intensify the MVA pathway for obtaining a high-yield FPP strain, designated E0. The CwGGPS1–5 genes were respectively cloned into the pET32a vector under the control of the T7 promoter and subsequently transformed into strain E0, resulting in strain E1. GGOH, the dephosphorylated derivative of GGPP, was measured to assess the catalytic activity of CwGGPSs in *E. coli*. After fermentation, GGOH was detected exclusively in the fermentation products of strain E1 harboring CwGGPS1, as determined by GC analysis, while no GGOH was produced in strains containing CwGGPS2-5 (**Supplementary Figure S1**). For further confirmation, GC-MS analysis identified the product (peak 1) from strain E1 (CwGGPS1) as GGOH, based on its retention time (RT = 9.988 min) and mass spectrum matching that of the GGOH standard (**Figures 4A, B**). Unexpectedly, trace amounts of GGOH were detected in the negative control strain E0. To further substantiate our findings, a quantitative analysis was carried out. The titer of GGOH in srain E1 (CwGGPS1) was found to be twice as high as that in the negative control srain E0 (**Figure 4E**; **Supplementary Figure S2A**). These results indicate that CwGGPS1 exhibits GGPS activity.

To investigate the catalytic activity of CwFPS1, the *ispA* gene in the plasmid pBbA5c-MevT (CO)-T1-MBIS (CO, ispA) was deleted to generate the mutant plasmid pBbA5cΔispA. The *ispA* gene encodes an enzyme responsible for the biosynthesis of FPP from DMAPP/GPP and IPP (Fujisaki et al., 1990; Zhou et al., 2015). The mutant plasmid pBbA5cΔispA was introduced into BL21star (DE3) to generate the E2 strain. Subsequently, CwFPS1 was introduced into the E2 strain to create the restored E3 strain. Although the ispA gene has been deleted from the plasmid pBbA5cΔispA, strain E2 can still produce FPP because the *ispA* gene is present in the genome of the E2 strain (Fujisaki et al., 1990). However, the production of FOH was significantly reduced in the E2 strain compared to that in the E0 strain. As predicted, the production was restored by CwFPS1, resulting in increased FPP levels in the E3 strain (**Figures 4C–F**; **Supplementary Figure S2B**). The results demonstrate that CwFPS1 exhibits FPS activity.

### 3.6 Enzymatic activity analysis of CwGGPSs and CwFPS1

**Figure 5:**
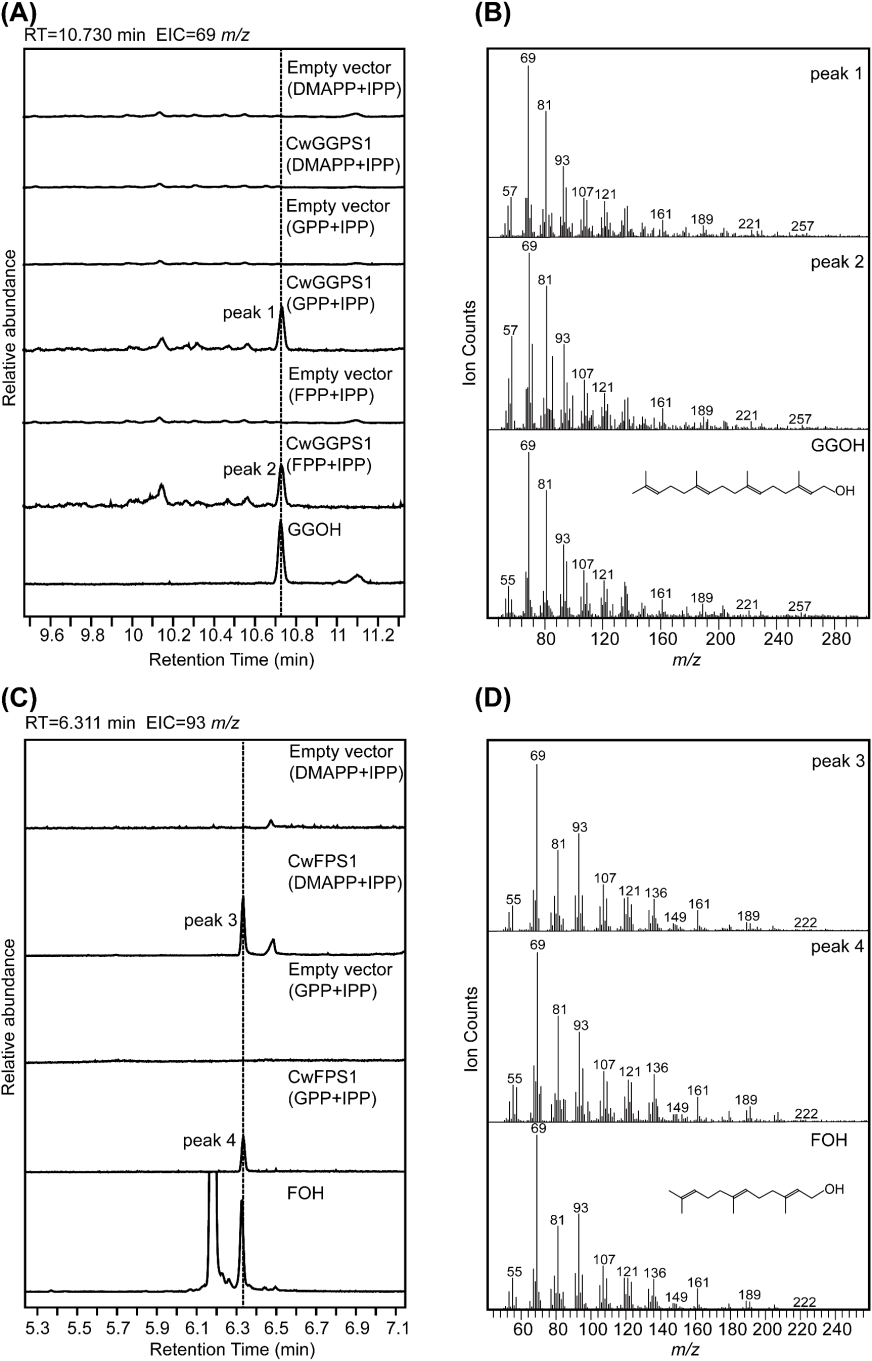
GC–MS analysis of the products catalyzed by CwFPS1 and CwGGPS1 *in vitro*. **(A)** GC–MS analysis of the dephosphorylated products catalyzed by CwGGPS1 using DMAPP/IPP, GPP/PP, and FPP/IPP as substrates. **(B)** Mass spectra of peak 1, peak 2, and GGOH. **(C)** GC–MS analysis of the dephosphorylated products catalyzed by CwFPS1 using DMAPP/IPP and GPP/IPP as substrates. **(D)** Mass spectra of peak 3, peak 4, and FOH.

To further confirm the enzymatic activity, purified CwFPS1 and CwGGPSs recombinant proteins were obtained from *E. coli* BL21 (DE3) (**Supplementary Figure S3**). Subsequently, enzymatic reactions were performed *in vitro*. The GC-MS analysis showed that GGOH (RT = 10.730 minutes) was produced from the substrates GPP/IPP or FPP/IPP through the catalytic action of CwGGPS1 (**Figures 5A, B**). However, GGOH was not observed when DMAPP/IPP were used as substrates. Additionally, no intermediate compounds, GOH (RT = 3.200 minutes) and FOH (RT = 6.311 minutes), were detected (**Supplementary Figure S4A**). FOH (RT = 6.311 min) was generated by CwFPS1 using both DMAPP/IPP and GPP/IPP as substrates (**Figures 5C, D**). Similarly, no intermediate compounds GOH (RT = 3.200 minutes) were observed (**Supplementary Figure S4B**). Furthermore, CwGGPS2, 3, 4, and 5 all lack catalytic activity to produce GOH, FOH, and GGOH when using DMAPP/IPP, GPP/IPP, and FPP/IPP as substrates (**Supplementary Figure S5**). These *in vitro* enzymatic reactions results were consistent with those obtained from heterologous expression in *E. coli*.

## 4 Conclusion

In this study, eight *trans*-CwPTs were identified and analyzed. As summarized in **Table 1**, CwPT1–5 were categorized into the GPS/GGPS/GFPS group, CwPT6 was assumed to be an FPS, while CwPT7 and CwPT8 were classified as SPS, based on evolutionary relationships and sequence structures. Furthermore, through heterologous expression in *E. coli* and *in vitro* enzymatic assays, it was confirmed that CwPT1 (CwGGPS1) and CwPT6 (CwFPS1) exhibit GGPS and FPS activities, respectively. In the future, we will further investigate the catalytic functions of *trans-*CwPTs with different allylic substrates. Additionally, the expression profile of *trans-CwPTs* was significantly induced by MeJA and ABA, indicating that MeJA and ABA may induce terpenoid biosynthesis. In the future, we will further elucidate the function of *trans-*CwPTs in *C. wenyujin*.

**Table 1:** Catalytic products of CwGGPS1 and CwFPS1 with different substrates *in vitro*.

Substrates	CwGGPS1	CwGGPS2	CwGGPS3	CwGGPS4	CwGGPS5	CwFPS1
DMAPP/IPP	–	–	–	–	–	FPP
GPP/IPP	GGPP	–	–	–	–	FPP
FPP/IPP	GGPP	–	–	–	–	

“-” indicates that there are no products available.

## Data Availability

The datasets presented in this study can be found in online repositories. The names of the repository/repositories and accession number(s) can be found in the article/**Supplementary Material**.
