# Supplementary material for: Systematic characterization and functional analysis of trans-prenyltransferases in Curcuma wenyujin
Source: Front Plant Sci. 2025 Nov 24;16:1712697. doi: 10.3389/fpls.2025.1712697 (PMC12682871; doi:10.3389/fpls.2025.1712697)
Supplement: Supplementary file 1 [file DataSheet1.docx]

Supplementary Material

# Table S1 The primer pairs used in this study

| Experiments | Gene name | Primer pairs (5’-3’) |
| --- | --- | --- |
| Gene Clone | CwGGPS1 | CTTCGCCACCGCCTTCTG |
|  |  | TCTGGCGGTAGGCTATGTAA |
|  | CwGGPS2 | TACGCCACCGCCGCTTGC |
|  |  | TACGCCACCGCCGCTTGC |
|  | CwGGPS3 | ATGGCTTCTTTGATGGTTCC |
|  |  | TCTGGCGGTAGGCGATGTAG |
|  | CwGGPS4 | GCCTTCTCCGCCTCTTTCTT |
|  |  | GTCTCCCTTTGAAAATGTTTCA |
|  | CwGGPS5 | ATGGCTTACGCTTCTGTTTG |
|  |  | TGGCGGTCGGCGATGTAG |
|  | CwFPS1 | ATGGCGGAGGCGACGGCGAA |
|  |  | TTACTTCTGCCTTTTGTAG |
|  | CwSPS1 | CCTCGCCTTTTCTCCACAT |
|  |  | CCATTCCATCTCCATTCAGCA |
|  | CwSPS2 | ATGTTCAGGTGGGGCTTCA |
|  |  | TTCGATCGATTGATCACCTTG |
| Expression profiles analysis | CwGGPS1 | GGTCAGCCCACCAACCACGTCGTCT |
|  |  | CCCCTCGCACGAAATGTCCACAATC |
|  | CwGGPS2 | GGACGACATCCTGGACGTGA |
|  |  | ACCAGCGTGTGAGCGAATT |
|  | CwGGPS3 | CAGTGACAAAACAACCTACCCAA |
|  |  | GGAGGAGATAACCTGTGCCATG |
|  | CwGGPS4 | AGGAGCTGGATAAGTTCAAGGAC |
|  |  | CTCAAACGAGCCAAAAGTGTTAC |
|  | CwGGPS5 | CGGGTTATGCTCAGGTGAG |
|  |  | GAGACGAGCAAGCAGTCCTCAC |
|  | CwFPS1 | CTTTCTTGGTGGTGTCCTTGG |
|  |  | GCTGTCTGAAACTCAACCTCGT |
|  | CwSPS1 | AAGTGGTGGGCTCAGGATGG |
|  |  | ATTTCGGAGGGCGGTTG |
|  | CwSPS2 | GCGCTTGTCGATCTAACTCACA |
|  |  | TGCTTAACAAATAGGAATAAATGTA |
|  | 18S | AACGAGACCTCAGCCTGCT |
|  |  | CCCAGAACATCTAAGGGCAT |
| Construction of prokaryotic expression vector | pET32a-CwGGPS1 | CTGATATCGGATCCCAGATGGCCTTCGCCACCGCCTTCTG |
|  |  | CTTGTCGACGGAGCTCTGGTTCTGGCGGTAGGCTATGTAA |
|  | pET32a-CwGGPS2 | CTGATATCGGATCCCAGATGGCCTACGCCACCGCCGCTTGC |
|  |  | GCTTGTCGACGGAGCTCTGGTTGTGGCGGGTGGCTATGAAG |
|  | pET32a-CwGGPS3 | GCTGATATCGGATCCCAGATGGCTTCTTTGATGGTTCC |
|  |  | TGTCGACGGAGCTCTGCTTCTGGCGGTAGGCGATGTAG |
|  | pET32a-CwGGPS4 | CTGATATCGGATCCCAGATGGCCTTCTCCGCCTCTTTCTT |
|  |  | CTTGTCGACGGAGCTCTGGTCTCCCTTTGAAAATGTTTCA |
|  | pET32a-CwGGPS5 | GCTGATATCGGATCCCAGATGGCTTACGCTTCTGTTTG |
|  |  | TGTCGACGGAGCTCTGGTTGTGGCGGTCGGCGATGTAG |
|  | pET32a-CwFPS1 | TGGCTGATATCGGATCCCAGATGGCGGAGGCGACGGCGAA |
|  |  | AGCTTGTCGACGGAGCTCTGCTTCTGCCTTTTGTAGATCT |
| Prokaryotic expression vectors of CwPTs | pMAL-CwGGPS1 | GAAGGATTTCAGAATTCGGAATGGCCTTCGCCACCGCCTTCTGCG |
|  |  | TGGTGGTGGTGGTGGTGGGAGTTCTGGCGGTAGGCTATGTAACGG |
|  | pMAL-CwGGPS2 | GAAGGATTTCAGAATTCGGAATGGCCTACGCCACCGCCGCTTGCT |
|  |  | TGGTGGTGGTGGTGGTGGGAGTTGTGGCGGGTGGCTATGAAGCGG |
|  | pMAL-CwGGPS3 | GAAGGATTTCAGAATTCGGAATGGCTTCTTTGATGGTTCCTTGTG |
|  |  | TGGTGGTGGTGGTGGTGGGACTTCTGGCGGTAGGCGATGTAGTTT |
|  | pMAL-CwGGPS4 | GAAGGATTTCAGAATTCGGAATGGCCTTCTCCGCCTCTTTCTTTT |
|  |  | TGGTGGTGGTGGTGGTGGGAGTCTCCCTTTGAAAATGTTTCACTG |
|  | pMAL-CwGGPS5 | GAAGGATTTCAGAATTCGGAATGGCTTACGCTTCTGTTTGCTTCC |
|  |  | TGGTGGTGGTGGTGGTGGGAGTTGTGGCGGTCGGCGATGTAGCGG |
|  | pMAL-CwFPS1 | GAAGGATTTCAGAATTCGGAATGGCGGAGGCGACGGCGAACGGAG |
|  |  | TGGTGGTGGTGGTGGTGGGACTTCTGCCTTTTGTAGATCTTTTCG |

**Table S2 The amino acid sequences of identified *trans*-CwPTs in *C. wenyujin***

| Name | Amino acid sequence |
| --- | --- |
| *Cw*GGPS1 | MAFATAFCVHGTNLATKPAARRGAAGSASRRPMVAVVRCVSTRKETGNLVVDQFDLQEYMAEKARKVNEALDLAVPIRHPEVIHRSMRHSLLAGGKRVRPLLAIASCELVGGDEKAAMPVACASEMIHTMSLIHDDLPCMDNDGLRRGQPTNHVVFGEDTAILAGDALLSFAFEHVAVSTVGVAPERVLRSIVELGNCVGSDGLVAGQIVDISCEGKVVDKDVLEYIHIHKTARLLEAAAVCGAIVGGGNDEEVDRIRSYARCVGLLFQVVDDILDVTKTSEELGKTAGKDLASDKTTYPKLLGLDGAREFAQSLVRKADGELAVFDAARAAPLYHLARYIAYRQN |
| *Cw*GGPS2 | MAYATAACSYAANMAAKPAARTAAAGAASRRPLAAAVHCVSTPPELGRLVVERFDLKEYMAEKARRVNEALEQAVPLRHPELIHRSMRHSLLAGGKRVRPILAIAACELVGGGEAAAMPVACAAEMIHTMSLIHDDLPCMDNDDLRRGQPTNHVLFGEDTAILAGDALLSFAFEHVAANAAGVAPERIVRAVLELGNSFGSEGLVAGQIVDISCEGKEVGMGVLEYIHLHKTARLLEAAAVCGAIMGGGEEAEVERVRRYARSVGLLFQVVDDILDVTKTSEELGKTAGKDLTSDKTTYPKLLGLDGAREFAHTLVRQAEGELAAFDAARAAPLYHLARFIATRHN |
| *Cw*GGPS3 | MASLMVPCGCCNAAISRVYLSGAAFRPAPPAVGFGSPIAIWAAHLSRRGPWLPRVMEVETSPAPPETVLSTGFDFKGYMLEKAAAVNRALDAAVPLAHPERIHEAMRYSLLAGGKRVRPVLCLASCEGVGGGSAWAMPAAVSVEMIHTMSLIHDDLPCMDDDDLRRGRPSCHRAFDEATAVLAGDALLALAFGHLANPASYPADGSVPPDRIVRAVGELSRRVGAEGLVAGQVADLEATGIGTPVTLDHLEFIHLHKTAALLEASVVLGAILGGASDDQIEKLRKYANSLGLLFQVVDDILDVTKSSEELGKTAGKDLASDKTTYPKLLGLEKSKEFADRLLKDSREQLAGFDPTKMAPLLHLANYIAYRQK |
| *Cw*GGPS4 | MAFSASFFYPSPKLSGTKMPPSPLLFRKPRSISLPVRCFSAAAVASFDLRTYWTTLISQIEEALDAAVPVRYPESIHKAIRHIVLSPGAKRAPPIMCIASCELVGGHRSAAFPAACALEMVHAASLVHDDLPCMDASPLRRGHPSAHALFGVDMAVLAGDALFPLAYQHIVTHTPSPDPVPLSIIPLVLTEIARAVGSTGMAAGQFLDLSDAAAGSEKEILQVLEKKFGEMAECSAVCGGLLGGASDQELEALRRYGRAVGVLYQLVDDVLMESNGGTGKMRSNASVVKAVGMDRALELVEEIRAKGKKELDKFKDKYGEKTLPLYSFVDYAVERGFFVEGLEAAAVAGGSDGSSETFSKGD |
| *Cw*GGPS5 | MAYASVCFPGYAQVRTACSSPSWGLAARPRRLLPAVRCASAAPEADLFDLKLYMAEKARRVDEALDRALPLRHPEPLLNAMRYSLLAPGKRVRPVLTLAACELVGGDESAAMPVACAAEMLHVMSLIHDDLPCIDNDDLRRGRPSNHAAFGEDVAVLAGDALHCFAFEHAADATAGVPPNRVLWAVAELAKATGSEGLPAGQVVDIESEGKEVGLEVLEYIHLHKTACLLEASVACGGIIGGAEDEEVERLRRYGRAVGLLFQVVDDVLDVTRSSEELGKTAGKDLANGKTTYPKLMGLDKARLLAEKLVAKAEEELQGFDRSRARPLRHLARYIADRHN |
| *Cw*FPS1 | MAEATANGAAAVAAGGDRKAAFLQIYVQLKEDLLRDPAFDYTDESREWIEKMLDYNVPGGKLNRGISVIDSYKLLKQGNQLTDTEFFLGGVLGWCIEWLQAYFLVLDDIMDNSVTRRGQPCWFRVPKVGLIATNDGILMRNHIPRMLKKYFKGKPYYVDLLDLFNEVEFQTASGQLLDLITTHEGEQNLSKYNITVYNRIVQYKTAYYSFYLPVACALLLAGKNLDNFLEVKDILIQMGTYFQIQDDYLDCFGHPDVIGKIGTDIQDFKCSWLVVQALGLANESQLKILSDNYGKTDPACVEKVKRLYKDLNLEKLFAEYERTSYGKLISSIEAQPGKEVQHVLKSFLEKIYKRQK |
| *Cw*SPS1 | MLSLTCPSVDLSQSGCLGRRRVRPLWRSRAARSAGARCMVSTTQDDVRSGGVATSVKPAVSSSGSIQVSSLLEVVSADIKRLNENLKALIGAENPVLVSAAEQIFGAGGKRLRPALVFLVSRATAQIAGLKELTVQHRRLAEIIEMIHTASLIHDDVIDDSGIRRGKETVHQIFGTRVAVLAGDFMFAQSSWYLANLENIEVIKLISQVIKDFASGEIKQASSLFNCDATLEDYLLKSYYKTASLVAASSKSASIFSGVDIAICERMYEYGKNLGLSFQIVDDILDFTQSTEQLGKPACSDLSKGNLTAPVIFALEKEPKLRKIIDSEFSEHGSFDTAIELIHQSGGLRMAQELAKQKAEVAIEILKCLPESEFKNSLEGIVKYNLERID |
| *Cw*SPS2 | MFRWGFRSLCRSRTAGSGVYSFNGFLPVASRGAHLSQKPAEVPSKVQDFREYHFQSSRGLHDIGYQIDQDRKYAAEESVDPFALVSDELSIIGNRLRSMVVSEVPKLASAAEYFFKIGAEGKRFRPTVLLLMASALSMPTPESVAAEVASSFEKNMRTRQQCIAEITEMIHVASLLHDDVLDDADTRRGIGSLNFVMGNKLSVLAGFLLSRACVALASLKNTEVVSLLATVVEHLVTGETMQMSANAEQRHSMEYYMLKTYYKTASLISNSCKAIALLAGQTTEVAMHAYNYGRNLGLAFQLIDDVLDFTGTTASLGKGSLSDIRHGIITAPILFAKEEFPELQSIIDKGFSNPANVEAALDYLGKSRGIERTRALAVEHANSAVEAIHALPKSDNEAVITSRRALVDLTHKVINRSK |

# Table S3 The characteristics of identified *trans*-CwPTs in *C. wenyujin*

| Names | Renames | Proteins  (aa) | MW  (kDa) | pI | Subcellular prediction | Predicted activity |
| --- | --- | --- | --- | --- | --- | --- |
| *Cw*PT1 | GGPS1 | 346 | 37.1 | 6.14 | Chloroplast | GGPP synthase |
| *Cw*PT2 | GGPS2 | 346 | 36.8 | 6.02 | Chloroplast | GGPP synthase |
| *Cw*PT3 | GGPS3 (LSU) | 372 | 39.6 | 6.01 | Chloroplast | GGPP synthase |
| *Cw*PT4 | GGPS4 (SSU-II) | 362 | 38.5 | 5.89 | Chloroplast | No activity |
| *Cw*PT5 | GGPS5 | 340 | 36.7 | 5.75 | Chloroplast | GPP/GGPP synthase |
| *Cw*PT6 | FPS1 | 356 | 40.7 | 5.74 | Cytoplasmic | FPP synthase |
| *Cw*PT7 | SPS1 | 390 | 42.5 | 6.36 | Chloroplast | long-chain SPP synthase |
| *Cw*PT8 | SPS2 | 418 | 45.9 | 6.65 | mitochondrion | long-chain SPP synthase |

# Table S4 The 150 *trans*-PTs used for construction of the phylogenetic tree

| **G(G)PS** | **FPPS** | **SPS** |
| --- | --- | --- |
| AAS82860.1 AmGPS | LeGGPPS1 Solyc11g011240 | AT1G78510 AtSPS1 |
| AAS82859.1 AmSSU | LeGGPPS2 Solyc04g079960 | AT1G17050 AtSPS2 |
| AAT71982.1 AtGGPPS10 | LeGGPPS3 Solyc02g085700 | AT2G34630 AtPPS |
| NP_189589.1 AtGGPPS9 | LeSSU-I Solyc07g064660 | BAF98298.1 HbGPS |
| AT3G14550 AtGGPPS7 | LeSSU-II Solyc09g008920 | ABD92707.1 HbSPS |
| AT3G14530 AtGGPPS6 | LeFPPS1 Solyc12g015860.1 | AFJ52721.1 MiGPS |
| AT3G20160 AtGGPPS8 | LeGPS Solyc08g023470.3 | AHL84161.1 NtGPS |
| At1g49530 AtGGPPS1 | LeSPS Solyc07g061990.3 | AEZ55677.1 SmGPS |
| At2g18620 AtGGPPS2 | AT5G47770 AtFPS1 | AAN86061.1 CuGPS |
| At2g18640 AtGGPPS3 | AT4G17190 AtFPS2 | CAO17862.1 Vv |
| At2g23800 AtGGPPS4 | ADJ67472.1 AaFPPS | ACA21459.1 PaGPS |
| At4g36810 AtGGPPS11 | AAP74719.1 AsFPPS | CAC20852.1 QrGPS |
| At4g38460 AtGGPPS12 | XP_009128999.1 BrFPPS | BAK05302.1 Hv |
| Os07g39270 OsGGPPS1 | CAA59170.1 Ca | BAK00672.1 Hv |
| Os01g14630 OsGPS | ADO95193.1 CrFPPS | Os06g46450 OsSPS1 |
| Os02g44780 OsSSU-II/OsGRP | XP_006474825.1 CsFPPS | Os05g50550 OsSPS2 |
| AGL91645.1 CrLSU | XP_008463060.2 CmFPPS | Os12g17320 OsSPS3 |
| AGL91646.1 CrGPS | AAM98379.1 HbFPPS | Os08g09370 OsSPS4 |
| AAS82870.1 CbSSU | BAB40665.1 HlFPPS | XP_002438852.1 SbSPS |
| ABW06960.1 CaGGPPS | AGQ04160.1 LaFPPS | XP_021321875.1 SbSPS |
| BAF98303.1 HbGGPPS | AAM08927.1 MdFPPS | XP_021321027.1 SbSPS |
| BAB60678.1 HbGGPPS | AFJ52720.1 MiFPPS | XP_008643450.1 Zm |
| BAF98300.1 HbGPS | ADC32809.1 MsFPPS | NP_001347618.1 ZmSPS |
| ACQ90682.1 HlLSU | XP_003594327.1 MtFPPS |  |
| ACQ90681.1 HlSSU | AAK63847.1 MpFPPS |  |
| AIY24421.1 JsGGPPS | AHM22931.1 NtFPPS |  |
| AFJ52722.1 MiGPS | AAY87903.1 PgFPPS |  |
| ADG01841.1 MsGGPPS | AAY53905.1 PnFPPS |  |
| AAF08793.1 MpLSU | ADJ68004.1 PqFPPS |  |
| AAF08792.1 MpSSU | ABV08819.1 SmFPPS |  |
| AFB35651.1 NtGGPPS3 | AAX76910.1 VvFPPS |  |
| NP_001312106.1 NtGGPPS | AAR27053.1 GbFPPS |  |
| ADD49735.1 NtGGPPS | ACA21460.1 PaFPPS |  |
| AEZ55680.1 SmSSU-II | AAS19931.1 TmFPPS |  |
| AEZ55681.1 SmLSU | ADV03674.1 ApFPPS |  |
| ACR19637.1 SmGGPPS | ADR83704.1 ApFPPS |  |
| AEZ55682.1 SmGGPPS2 | AER12202.1 HcFPPS |  |
| AEZ55683.1 SmGGPPS3 | AFP19446.1 CgFPPS |  |
| AEZ55678.1 SmSSU-I | AFX68799.1 DoFPPS |  |
| AEZ55679.1 SmSSU-II | BAJ84778.1 Hv |  |
| CAA67330.1 SaGGPPS | BAJ90047.1 Hv |  |
| CAO38946.1 Vv | BAJ87514.1 Hv |  |
| CAO64763.1 Vv | ADZ57167.1 LlFPPS |  |
| AAN01133.1 AgGPS | AHA51120.1 AbFPPS |  |
| AAN01134.1 AgGPS | Os01g50760 OsFPS1 |  |
| AAN01135.1 AgGPS | Os05g46580 OsFPS2 |  |
| ACO59905.1 EuGGPPS | Os01g50050 OsFPS3 |  |
| AAQ72786.1 GbGGPPS | Os04g56230 OsFPS4 |  |
| ADD82422.1 JcGGPPS | Os04g56210 OsFPS5 |  |
| ACA21458.2 PaGPS | XP_008792798.2 PdFPPS |  |
| ACZ57571.1 PaIPS | XP_002456217.1 SbFPPS |  |
| AGU43761.1 PmGGPPS | XP_002441458.1 SbFPPS |  |
| AAS67008.1 TmGGPPS | XP_021319712.1 SbFPPS |  |
| BAJ94484.1 Hv | AFV51836.1 TaFPPS |  |
| BAJ96219.1 Hv | AGC11812.1 TaFPPS |  |
| XP_021311468.1 SbGGPPS7] | ACR35378.1 Zm |  |
| XP_002463084.1 SbGGPPS | ACF88176.1 Zm |  |
| XP_002452775.2 SbSSU |  |  |
| SPT17215.1 Ta |  |  |
| ABQ85648.1 ZmGGPPS3 |  |  |
| NP_001183930.1 ZmGGPPS1 |  |  |
| XP_008678927.1 ZmSSU |  |  |

**
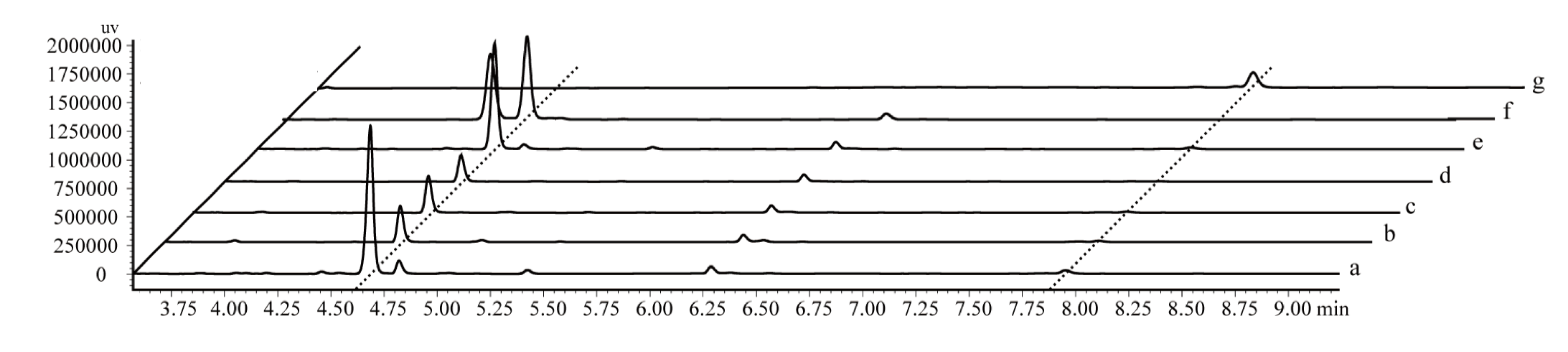
**

Figure S1 GC analysis of the products catalyzed by CwGGPS2-5 in *E. coli*. (a) strain E0. (b-e) strain E0 harbors CwGGPS2, CwGGPS3, CwGGPS4, and CwGGPS5, respectively. (f) FOH standard chemical compound. (g) GGOH standard chemical compound.


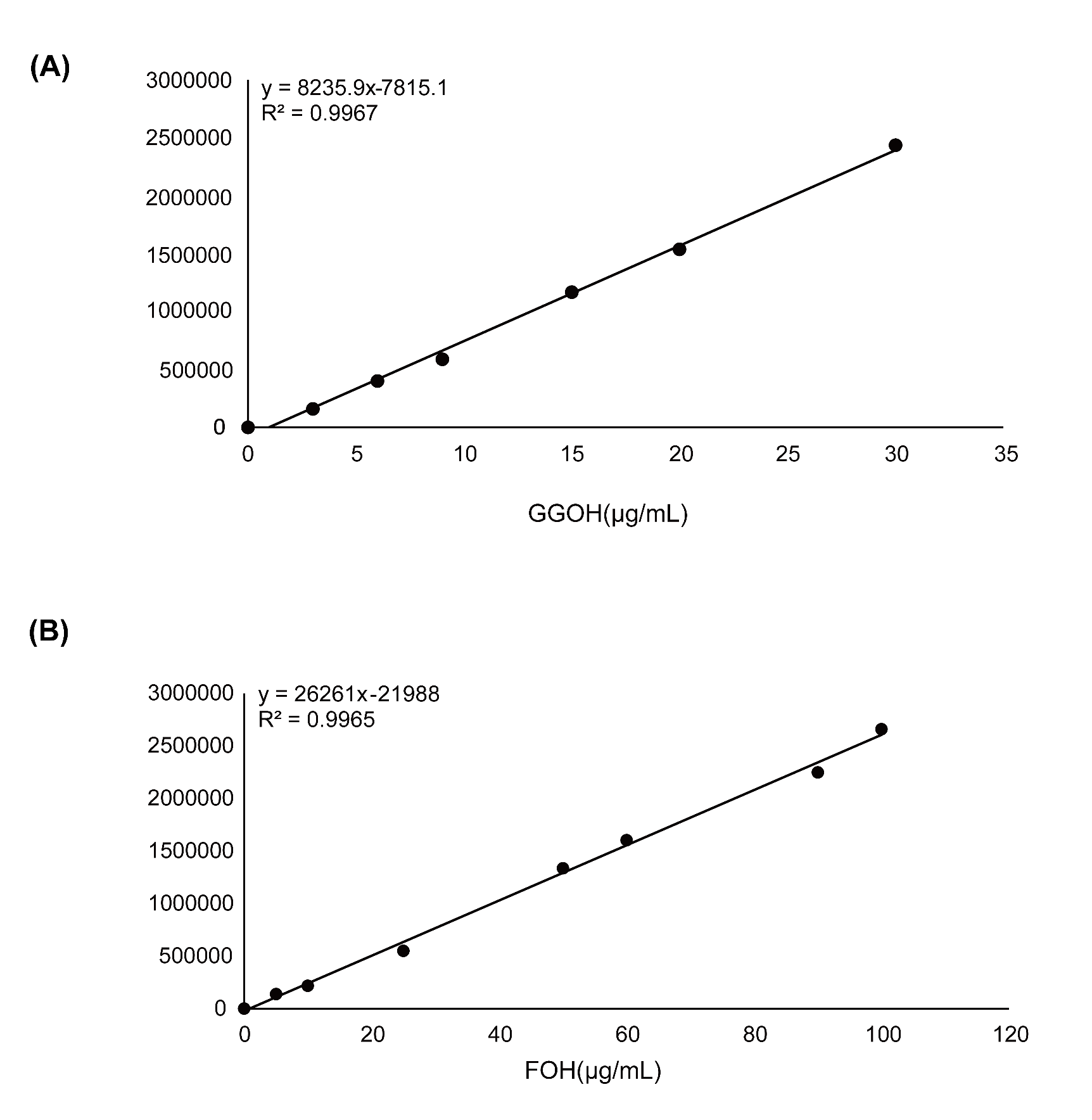


Figure S2 The standard curves of GGOH (A) and FOH (B).


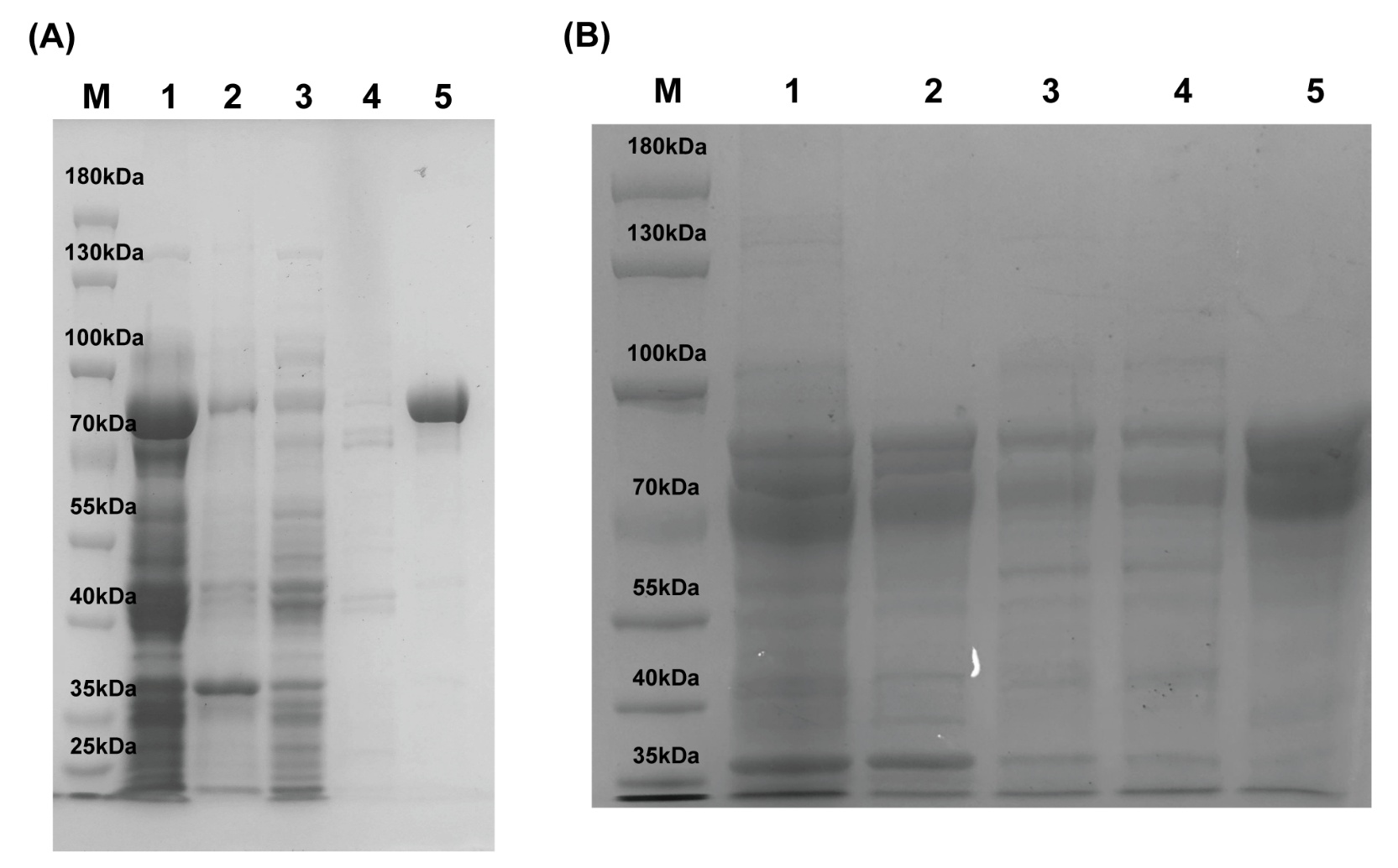


Figure S3 SDS-PAGE analysis of recombinant protein from *Escherichia coli* BL21 (DE3) harboring (A) pMAL-CwFPS1 and (B) pMAL-CwGGPS1. M, protein marker; 1, the soluble protein; 2, the insoluble protein; 3, flow-through solution; 4, washing solution; 5, purified CwFPS1 or CwGGPS1 recombinant protein.


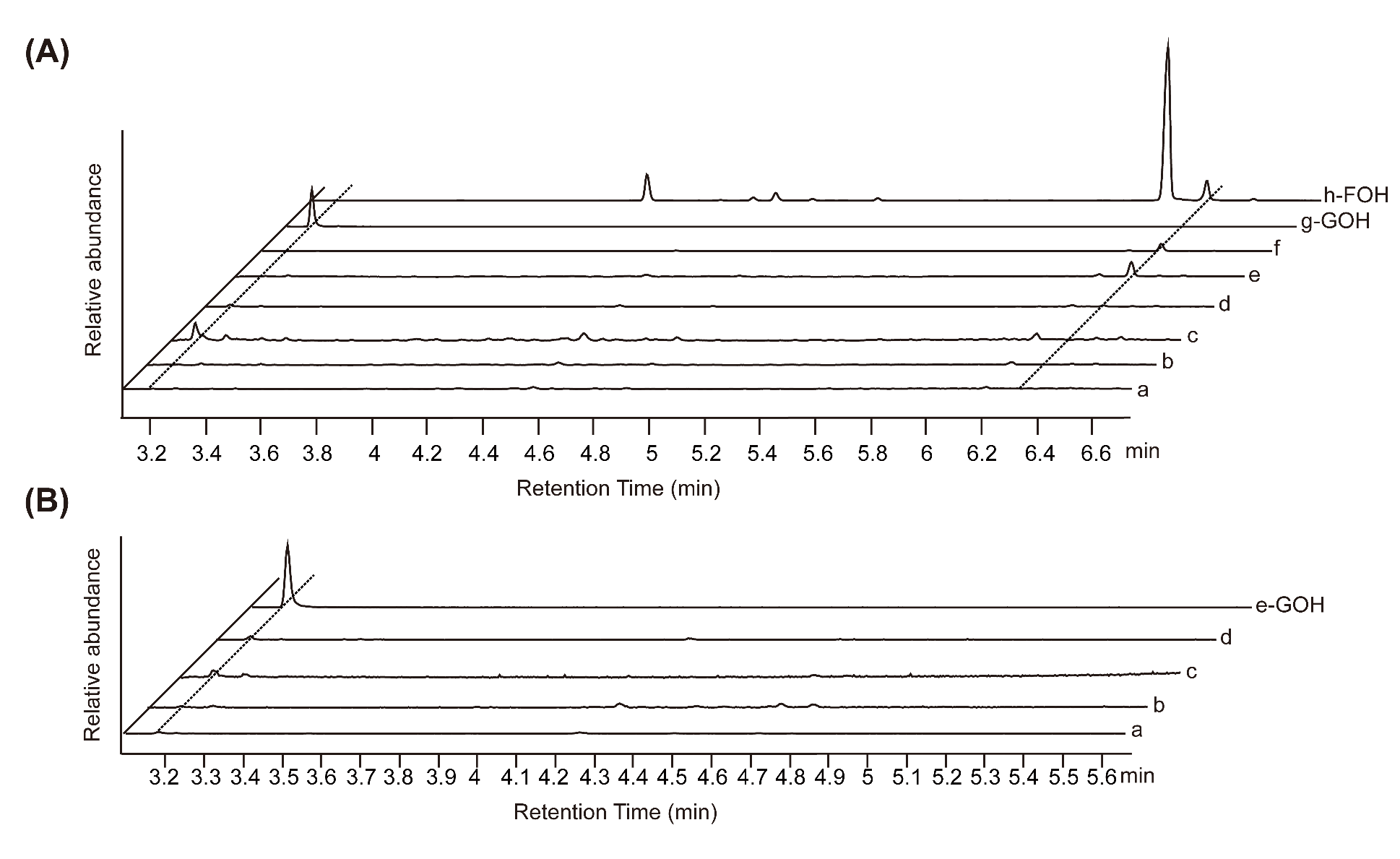


Figure S4 GC-MS analysis of the products catalyzed by CwGGPS1 andCwFPS1 *in vitro*. (A) the catalytic product of purified CwGGPS1 with different substrates, including CK-DMAPP and IPP (a), CwGGPS1-DMAPP and IPP (b), CK-GPP and IPP (c), CwGGPS1-GPP and IPP (d), CK-FPP and IPP (e), CwGGPS1-FPP and IPP (f), GOH standard chemical compound (g), FOH standard chemical compound (h). (B) the catalytic product of purified CwFPS1 with different substrates, including CK-DMAPP and IPP (a), CwFPS1-DMAPP and IPP (b), CK-GPP and IPP (c), CwFPS1-GPP and IPP (d), GOH standard chemical compound (e).


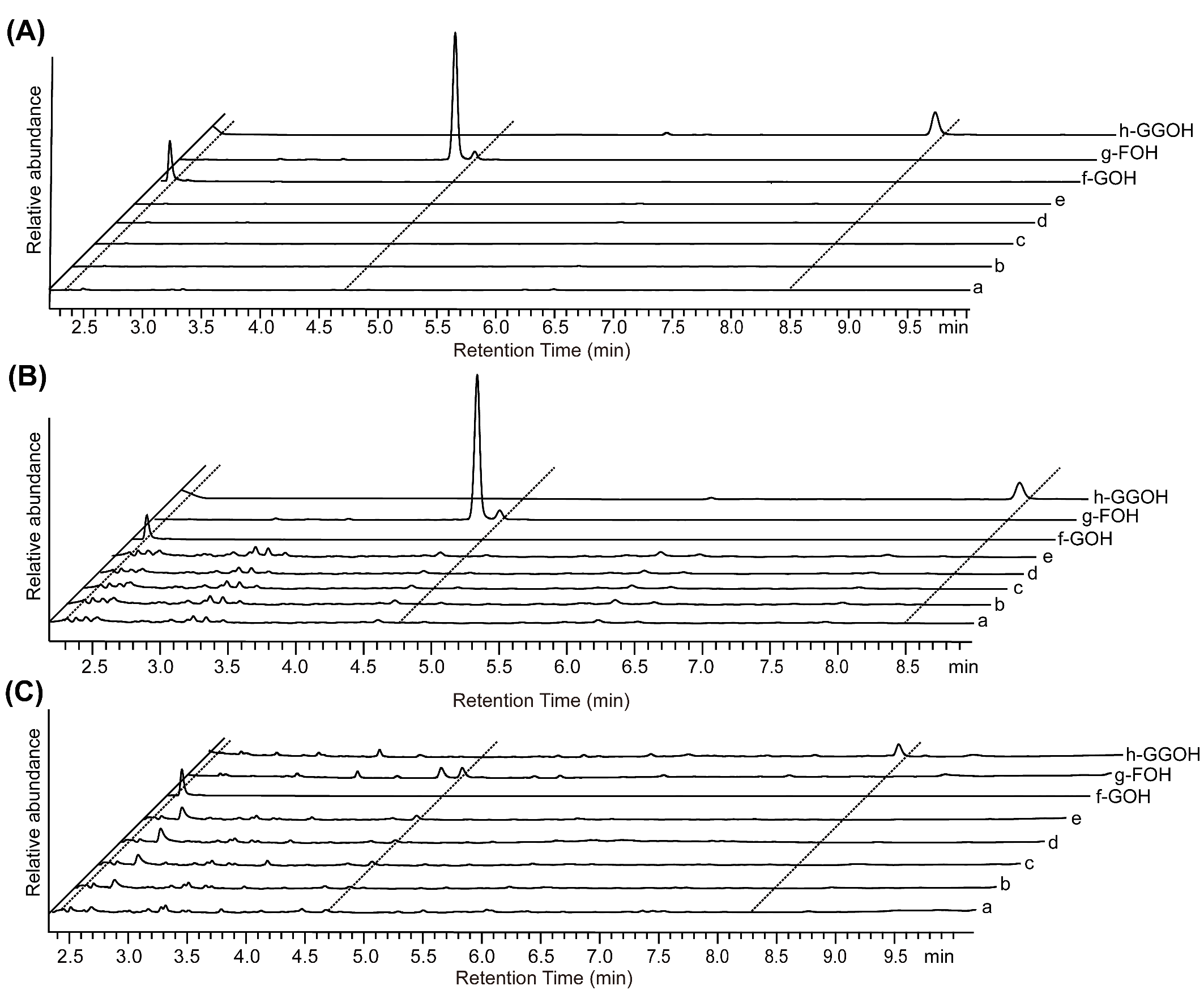


Figure S5 GC analysis of the products catalyzed by CwGGPS2-5 *in vitro*. (A) Substrates: DMAPP and IPP, (B) Substrates: GPP and IPP, (C) Substrates: FPP and IPP, (a) CK, (b) CwGGPS2, (c) CwGGPS3, (d) CwGGPS4, (e) CwGGPS5 (f) GOH standard chemical compound, (g) FOH standard chemical compound, (h) GGOH standard chemical compound.
